# Psychological Determinants of Attitude to Surgery in Internal Carotid Artery Stenosis Patients

**DOI:** 10.3390/healthcare9060775

**Published:** 2021-06-21

**Authors:** Michał-Goran Stanišić, Teresa Rzepa, Przemysław Kubaszewski

**Affiliations:** 1 Department of Vascular and Endovascular Surgery, Angiology and Phlebology, Poznan University of Medical Sciences, 61-848 Poznań, Poland; 2 University of Social Sciences and Humanities, 61-719 Poznań, Poland; trz@data.pl (T.R.); kubaszewski@gmail.com (P.K.)

**Keywords:** personal resources, internal carotid artery stenosis, stroke, psychological determinants

## Abstract

The basic way to prevent cerebral stroke in symptomatic 70–99% stenosis of internal carotid artery (ICA) is an open or endovascular surgical procedure. Psychological research done so far among ICA stenosis patients focused on cognitive functioning changes. The objective was to assess attitude to surgery in relation to self-efficacy, life quality perception, and health locus of control in ICA stenosis patients. Materials and Methods: The study involved 53 asymptomatic ICA stenosis patients, aged from 53 to 81. Four scales were applied: Generalized Self-Efficacy Scale (GSES); Satisfaction With Life Scale (SWLS); Multidimensional Health Locus of Control Scale (MHLC); and a simple scale to examine the attitude to surgery, where “−10” stands for the maximally negative attitude, “0”—neutral, and “+10”—maximally positive. The obtained results were put to statistical analysis. Results: It was found that women and men assessed their attitude to the surgery as positive (*M* = 7.92; *SD* = 3.094), though the men estimated it slightly higher (*M* = 8.03; *SD* = 3.02) than the women (*M* = 7.67; *SD* = 3.37). The mean value of self-efficacy was high (*M* = 32.53; *SD* = 6.231), and slightly higher for the men (*M* = 32.79; *SD* = 5.576) compared to the women (*M* = 31.87; *SD* = 7.836). The patients generally tended to manifest the external personal health locus of control (*M* = 28.62; *SD* = 3.17). The runner-up was internal health locus of control (*M* = 26.02; *SD* = 3.775), and the next one—external impersonal aspect (chance/luck) (M = 23.57; SD = 4.457). The mean assessment of the patients’ own life quality proved to be above average (*M* = 23.60) but varied (*SD* = 5.95). The women perceived the quality of their lives as better (*M* = 24.33; *SD* = 6.422) than the men (*M* = 23.32; *SD* = 5.818). Very strong positive correlations were found between self-efficacy and life quality assessment (*p* < 0.001) and between the internal and external personal aspects of health locus of control (*p* < 0.007) in the women, and positive correlations were found between the attitude to surgery and internal health locus of control (*p* < 0.021) in the men. Conclusions: When breaking the news of a need to have a surgical intervention due to ICA stenosis, the physician should strongly refer to the value of human life and health. The message should arise from external (in the case of women) or internal (in the case of men) motivation to undergo surgery, and enhance the patient’s conviction that the disease should be considered a challenge which must be taken to reverse their unfavorable situation and improve life quality.

## 1. Background 

Internal carotid artery stenosis (ICAS) is a recognized risk factor for cerebral stroke. Apart from pharmacological treatment, the basic way to prevent cerebral stroke is an open or endovascular surgical procedure [1]. Although a standard procedure in symptomatic internal carotid artery stenosis is open endarterectomy, there are still pending discussions about whether interventions are advisable in asymptomatic internal carotid artery stenosis and what kind of intervention to choose [2]. In light of the current research results, a surgical intervention for asymptomatic ICAS is justified when the stenosis is 70–99% [3,4]. Despite the documented beneficial effects of surgical treatment for the patients’ population, the individual risk in elective surgery is still high and, according to different sources of data, the cumulative risk of cerebral stroke, cardiac infarction and death may reach 1.5–6% regardless of the method applied: endarterectomy (CEA) or stenting (CAS) [5,6]. Qualifying an ICAS patient for surgical treatment requires the physician not only to understand the nature of the condition and the surgical treatment benefits for the patient, but also to know how the patient may react to the news of the need for surgery in the case of an asymptomatic condition. 

Psychological research done so far among ICA stenosis patients focused on cognitive functioning changes, measured mainly after the surgery [7,8,9,10,11]. However, there are few studies on the requirements for a risky decision to be taken by patients who find themselves in a difficult situation in life [12,13]. Taking a decision to undergo surgery and developing a positive attitude to it requires activation of personal resources that allow the patient to cope with the sudden news of a fatal disease [14,15]. The resources include self-efficacy and health locus of control, as they ease the patients to manage the stressful situation related to the disease. The adaptation may be reinforced by a positive perception of patients’ life quality. Previous research in the field of cardiovascular surgery shows fluctuations in health locus of control in peri-operative phases, requiring additional studies on specific disorders [16].

Self-efficacy is defined as a presumption that one is able to meet one’s objectives, i.e., to make good decisions and cope with one’s tasks even in unexpected and stressful situations [17,18]. Health locus of control is defined as an expectation that the present state is a result of external factors or one’s own activities and exerting personal control [19]. Therefore, internal health locus of control is related to personal responsibility for a patient’s health. Health locus of control may also be external: a subject may have the feeling of dependency on other people (e.g., health care providers) or on fate or chance [20,21]. Life quality assessment, in turn, is subject to dynamic changes and is based on the aggregated evaluation of physical health, general mental condition, status of relations with surrounding people, and fulfillment in their social and occupational roles [22,23].

The described constituents of personal resources underlie the mechanisms of coping with life challenges, which in the case of ICAS patients means unexpected initial news of a potentially serious disease and the need to undergo an invasive procedure. 

The aim of the study was to assess attitude to surgery in relation to self-efficacy, life quality perception, health locus of control, and correlations between age, gender and attitude to surgery, and the above-mentioned constituents of personal resources in ICAS patients.

## 2. Methods

Consent was granted by the Research Ethics Committee; 53 asymptomatic ICA stenosis patients were recruited in a period of 6 months. The conditions of eligibility for the study were: asymptomatic disease for at least one year, elective admission, and the patient’s consent to participate in the study in the pre-operative period. The examination involved persons admitted to a hospital and meeting the aforementioned criteria during psychologist visits held twice a month. During the visit, the psychologist was able to assess from 2 to 6 patients scheduled for carotid intervention for the next day or the day after. Such a system allowed for examination of about 50% of the weekly load of asymptomatic carotid patients treated in the department. Within the study period, two patients refused to take part in the assessment and were not included in the study population. Asymptomatic patients were chosen to eliminate the bias of emergency pressure from health care professionals in the case of symptomatic carotid disease. Before admission, patients had at least two scheduled consultations with a vascular surgeon and were subjected to two independent duplex scans of carotid arteries or one duplex assessment followed by angio-MRI or CT. The patients were not differentiated in terms of the kind of planned surgery (CAS/CEA) due to the comparable cumulative risk for both procedures [5].

The researching psychologist applied 4 scales, which were completed—with the help of the researcher—by the patients awaiting elective interventions in the university vascular surgery department. The tests, completed in the afternoon after regular medical assessment, were:

1. Generalized Self-Efficacy Scale (GSES, by R. Schwarzer and M. Jerusalem; adapted by Z. Juczyński), composed of 10 statements and measuring the level of general personal conviction regarding the individual’s efficacy in handling tough situations [24];
2. Satisfaction With Life Scale (SWLS, by E. Diener, R.A. Emmons, R.J. Larson, and S. Griffin; adapted by Z. Juczyński), composed of 5 statements and measuring the generalized feeling of satisfaction with life so far [24];
3. Multidimensional Health Locus of Control Scale (MHLC, by K.A. Wallston, B.S. Wallston, and R. De Vellis; adapted by Z. Juczyński), composed of 18 statements and aimed at specifying their expectations in three aspects of health locus of control [24]. Thus, the study participant indicates whether their own health is controlled: (1) by them (internal aspect); (2) by other individuals, especially health professionals (external personal aspect); and (3) by fate/chance (external impersonal aspect);
4. A simple scale was applied to find the attitude to surgery, where “−10” stands for the maximally negative attitude, “0”—neutral, and “+10”—maximally positive.

Tests 1, 2, and 3 assessed mental dispositions of personal resources (self-efficacy, health locus of control, and life quality assessment), and test 4 assessed the type and intensity of patients’ attitude to surgery.

The statistical analysis of the results was done by means of IBM SPSS Statistics 22 software (IBM, Armonk, NY, USA). To assess the normality of distribution of the analyzed variables, the Shapiro−Wilk test was applied. In order to specify the strength of correlation between the variables, Pearson correlation coefficient (r) was applied. The level of the anticipated impact of variables was verified by means of regression analysis in the single-and multivariable model.

## 3. Results

The study population consisted of 53 patients aged from 53 to 81 (*M =* 66.94; *SD =* 8.05) who were recruited to the study and examined. The studied group included 15 women aged from 59 to 81 (*M =* 69.2; *SD =* 6.75) and 38 men aged from 53 to 81 (*M =* 66.05; *SD =* 8.42).

It was found that the women and the men assessed their attitude to the surgery as positive (*M =* 7.92; *SD =* 3.094), though the men estimated it slightly higher (*M* = 8.03; *SD* = 3.02) than the women (*M* = 7.67; *SD* = 3.37). The mean value of self-efficacy was high (*M* = 32.53; *SD* = 6.231), and slightly higher for the men (*M* = 32.79; *SD* = 5.576) compared to the women (*M* = 31.87; *SD* = 7.836). The patients generally tended to manifest the external personal health locus of control (*M* = 28.62; *SD* = 3.17). The runner-up was internal health locus of control (*M* = 26.02; *SD* = 3.775), and the next one—external impersonal aspect (chance/luck) (*M* = 23.57; *SD* = 4.457).

The mean assessment of life quality proved to be above average (*M* = 23.60), but varied (*SD* = 5.95). The women perceived the quality of their lives better (*M* = 24.33; *SD* = 6.422) than the men (*M* = 23.32; *SD* = 5.818).

The correlations between attitude to surgery, age, and gender proved to be statistically insignificant in relation to the totality of the patients. There were positive correlations between attitude to surgery and internal health locus of control (*p <* 0.004) and their own life quality assessment (*p <* 0.022). It was found that the variability of the internal health locus of control factor explained 13.4% of the patients’ attitude to surgery variability (*F*(1.51) = 9.08; *p <* 0.004).

Positive, statistically significant correlations were found between internal and external personal health locus of control (*p <* 0.002), between self-efficacy and life quality assessment (*p <* 0.000), between life quality assessment and external personal health locus of control (*p <* 0.037), and also between external personal and impersonal health locus of control (*p <* 0.028) (Table 1).

The attitude to surgery taken by the female patients did not depend on their age, but it showed a strong positive correlation with external personal health locus of control (*p <* 0.043). Besides, it was found that the variability of this factor (external personal health locus of control) explained 22.5% of the variability in female patients’ attitude to surgery (*F*(1.13) = 5.06; *p <* 0.043).

Moreover, very strong positive correlations were found between self-efficacy and life quality assessment (*p <* 0.001) and between internal and external personal health locus of control (*p <* 0.007) (Table 2).

Among the male patients, no statistically significant correlations were found between age and attitude to surgery. However, there were positive correlations between attitude to surgery and internal health locus of control (*p <* 0.021). Besides, it was also found that the variability of this factor (internal health locus of control) explained 11.6% of the variability in male patients’ attitude to surgery (*F*(1.51) = 5.86; *p <* 0.021).

Positive correlations were also found between internal and external personal health locus of control (*p <* 0.035), between external personal and impersonal health locus of control (*p <* 0.034), and between self-efficacy and life quality assessment (*p <* 0.049) (Table 3).

Men gave higher notes (*M* = 8.03) than women (*M* = 7.67) not only to their attitude to surgery, but also to their self-efficacy (respectively: *M* = 32.79 and *M* = 31.87).

## 4. Discussion

The study results prove that in the case of internal carotid artery stenosis (ICAS) patients who suddenly, during out-patient consultation, face the news of the disease and are required to make a tough and risky decision on surgery, it is necessary to pay particular attention to their personal resources—particularly with regard to their mental dispositions showing a correlation with positive attitude to surgery, as an optimistic attitude has a positive influence on the treatment and recovery process [25,26,27], and in the case of this disease, on taking a decision to undergo surgery [28]. After all, not only asymptomatic ICAS patients, but even some physicians find it difficult to estimate the cerebral stroke risk for undergoing or not undergoing the surgical operation, due to ongoing discussion over asymptomatic carotid artery stenosis. Patients estimated the cerebral stroke risk following surgery to be even as much as 65% [12]. Other studies [13] showed that the higher the estimated risk was, the older the patients were, the more operations they had undergone in their adult years, the more often they did not have social support and the less trust they had in the medical staff (low results in the area of external personal health locus of control).

Based on the research study, we may assume that the more positive the attitude to surgery, the lower the level of the related, estimated risk. While risk estimation is a complicated task, depending on many factors, it is easier to influence the variables determining the attitude to surgery (in terms of its kind and intensity). Therefore, it was interesting to check what psychological resources were activated by the patients who formed and showed positive attitudes to surgery. It was established that—regardless of age and gender [9,13]—patients were more positive the more they relied on personal control of their health and took responsibility for their health, and the more they valued their own life quality (Table 1). Besides, the variability of the major factor (internal health locus of control) explained 13.4% of the variability in attitude to surgery. This finding justifies formulating an important recommendation for physicians. Namely, they should talk to asymptomatic ICAS patients in such a way as to convince them of the possibility to take an independent and internally motivated decision on undergoing the surgery. Then, the news of the disease will be treated as a challenge that will activate the patient’s personal resources and will induce the patient to take an effective decision [27,28,29]. A similar recommendation was formulated as a conclusion drawn from the comparative studies regarding internal carotid artery stenosis (ICAS) and abdominal aorta aneurysm (AAA) patients [29,30].

The correctness of the above-mentioned recommendation is confirmed by other findings, as it was found that the higher the level of internal health locus of control, the greater the trust in medical professionals (Table 1) [7] and the greater the faith in having good luck in the current life situation (Table 1). The correlation between these factors results in growing self-efficacy correlated with a higher assessment of life quality (*p <* 0.000) [13,28,29].

Although the above-described correlations and the recommendation addressed to physicians pertain to the totality of patients, they involve some gender-related subtleties. Namely, the observed correlations were mainly influenced by the results obtained from the ICAS male patients. Although the number of female patients involved in the study was small, it was found that their attitudes to surgery showed a strong positive correlation with external personal health locus of control (Table 2). This means that the women’s attitudes are significantly dependent on information provided by and behavior of medical professionals [29]. Besides, it is worth noting that this finding confirms the common stereotype that women are more prone to suggestion. Since the variability of this factor explained as much as 22.5% of the variability of attitude to surgery, and since this significant result obtained by the female patients changed the overall sequence of aspects of health locus of control in favor of the external personal aspect, the recommendations for physicians communicating with ICAS female patients seem obvious. The more suggestive the communications used by physicians who—as it came out—constitute a strong external source of motivation for ICAS female patients, the bigger the chance to form positive attitudes to surgery [12,13,28]. The importance of this kind of motivation is proved by detailed statistical analyses showing that the greater the trust of ICAS female patients in medical professionals, the higher their self-efficacy in connection with a higher assessment of their own life quality (Table 2) and the greater the internal health locus of control (Table 2).

The above-mentioned gender differences were reflected in descriptive statistics, as it turned out that the men gave higher notes (*M* = 8.03) than the women (*M* = 7.67) not only to their attitude to surgery, but also to their self-efficacy (respectively: *M* = 32.79 and *M* = 31.87), even though the results were not reflected in assessment of their own life quality, which was more appreciated by the women (respectively: *M* = 23.32 and *M* = 24.33). These findings may also be explained by referring to the prevailing stereotypic perception of gender roles in the society. As men (according to the stereotype) “should be able to cope in each situation”, self-efficacy seems to fit them more. Women, in turn (according to the stereotype) [31], because of playing socially desired functions, “should feel happy, as they are badly needed”, and therefore should highly assess their own life quality.

Our study is limited to a specific disorder where the risk of ischemic stroke is the risk related to the disease and to the procedure. We assessed only asymptomatic patients and the researcher was present during the assessment creating a possible bias during specific test explanations.

## 5. Conclusions

Communicating the news of internal carotid artery stenosis, the physician should strongly refer to the value of human life and health, regardless of the gender and age of the patient.

The message should arouse external (in the case of women) or internal (in the case of men) motivation to undergo interventional treatment and reinforce the patient’s conviction that the disease should be treated like a challenge, so as to change their unfavorable life situation and improve their life quality.

## Figures and Tables

**Table 1:** Correlations between attitude to surgery, age, gender, and the selected constituents of personal resources in ICAS patients.

Variables	Age	Gender	Internal Health Locus of Control	External Personal Health Locus of Control	External Impersonal Health Locus of Control	Self-Efficacy	Life Quality Assessment
Gender	−0.178						
	0.203						
Internal health locus of control	−0.041	−0.154					
	0.770	0.272					
External personal health locus of control	0.008	0.031	0.415; *p <* 0.01				
	0.954	0.824	0.002				
External impersonal health locus of control	0.123	0.052	0.235	0.301; *p <* 0.05			
	0.380	0.711	0.091	0.028			
Self-efficacy	0.003	0.067	−0.085	0.086	0.034		
	0.986	0.632	0.543	0.539	0.809		
Life quality assessment	0.116	−0.078	0.267	0.288; *p <* 0.05	0.107	0.471; *p <* 0.01	
	0.410	0.580	0.053	0.037	0.448	0.000	
Attitude to surgery	0.153	0.053	0.389; *p <* 0.01	0.262	0.074	0.202	0.314; *p <* 0.05
	0.275	0.707	0.004	0.058	0.597	0.148	0.022

**Table 2:** Correlations between attitude to surgery, age, and the selected constituents of personal resources in ICAS female patients.

Variables	Age	Internal Health Locus of Control	External Personal Health Locus of Control	External Impersonal Health Locus of Control	Self-Efficacy	Life Quality Assessment
Internal health locus of control	−0.229					
	0.411					
External personal health locus of control	0.079	0.666; *p <* 0.01				
	0.781	0.007				
External impersonal health locus of control	−0.478	0.414	0.107			
	0.071	0.125	0.704			
Self-efficacy	−0.157	−0.110	0.010	−0.033		
	0.575	0.696	0.973	0.908		
Life quality assessment	−0.091	0.319	0.380	−0.120	0.756; *p <* 0.01	
	0.748	0.247	0.163	0.669	0.001	
Attitude to surgery	0.129	0.458	0.529; *p* < 0.043	0.054	0.252	0.457
	0.648	0.086	0.043	0.848	0.365	0.087

**Table 3:** Correlations between attitude to surgery, age, and the selected constituents of personal resources in ICAS male patients.

Variables	Age	Internal Health Locus of Control	External Personal Health Locus of Control	External Impersonal Health Locus of Control	Self-Efficacy	Life Quality Assessment
Internal health locus of control	−0.015					
	0.930					
External personal health locus of control	−0.002	0.343; *p <* 0.05				
	0.989	0.035				
External impersonal health locus of control	0.270	0.198	0.345; *p <* 0.05			
	0.101	0.233	0.034			
Self-efficacy	0.089	−0.055	0.121	0.057		
	0.596	0.742	0.470	0.734		
Life quality assessment	0.170	0.230	0.264	0.184	0.321; *p <* 0.05	
	0.307	0.164	0.110	0.269	0.049	
Attitude to surgery	0.180	0.374; *p <* 0.05	0.169	0.079	0.169	0.255
	0.280	0.021	0.309	0.639	0.310	0.122

## Data Availability

Original data sets are in disposition of co-authors of the manuscript.
